# Online Hemodiafiltration: A New Perspective for Patients With End-Stage Renal Disease

**DOI:** 10.7759/cureus.66076

**Published:** 2024-08-03

**Authors:** Diana D Nenova, Gergana M Chausheva, Yanko G Yankov

**Affiliations:** 1 Clinic of Nephrology and Dialysis, University Hospital "St. Marina", Varna, BGR; 2 Second Department of Internal Disease, Medical University "Prof. Dr. Paraskev Stoyanov", Varna, BGR; 3 Central Clinical Laboratory, University Hospital "St. Marina", Varna, BGR; 4 Department of Clinical Laboratory, Medical University "Prof. Dr. Paraskev Stoyanov", Varna, BGR; 5 Clinic of Maxillofacial Surgery, University Hospital "St. Marina", Varna, BGR; 6 Department of General and Operative Surgery, Medical University "Prof. Dr. Paraskev Stoyanov", Varna, BGR

**Keywords:** intradialytic hypotension, hemodialysis, quality of life, end-stage renal disease, survival, clinical outcome, clearance, middle molecules, convective therapies, online hemodiafiltration

## Abstract

Introduction

Online hemodiafiltration (OL-HDF) is the most effective renal replacement therapy (RRT), which allows the enhanced removal of small and large uremic toxins by combining diffusion and convective transport of solutes. Although the goal of OL-HDF is to provide greater clearance of solutes with a preference for intermediate molecules responsible for many of the complications of chronic kidney disease (CKD), the studies reported to date and their meta-analyses are conflicting in nature and do not show a significant advantage of convective therapies on patient prognosis.

Materials and methods

At the Clinic of Nephrology and Dialysis, University Hospital "St. Marina", Varna, Bulgaria, 41 patients were monitored in a retrospective study for a two-year period, randomized into two groups, conducting OL-HDF after dilution and hemodialysis (HD) with the aim of studying the effect of convective therapies on the clinical outcome, the achieved quality of life, and the prognosis of the patient.

Results

The study found a significantly higher quality of life in patients undergoing OL-HDF with significantly higher values ​​of indicators of dialysis adequacy and nutritional status, better control of the anemic syndrome with the reduction of erythropoietin doses, significantly lower frequency of episodes of intradialytic hypotension with improved recovery, and 3.6-fold lower risk of death compared with conventional dialysis.

Discussion

Three major randomized controlled trials have compared survival outcomes in patients receiving HD or post-dilution OL-HDF, reporting conflicting results. Meta-analyses of the published studies have also been unable to provide a clear and definitive answer regarding the potential benefits of choosing one treatment over the other. Overall mortality, anemia, phosphate control, and small molecule clearance appear to be insufficiently influenced by the treatment method. On the other hand, cardiovascular mortality, hemodynamic stability, and clearance of middle and protein-bound molecules seem to be better in patients treated with OL-HDF.

Conclusions

Despite the conflicting data reported so far, OL-HDF is associated with better clinical outcome and prognosis for end-stage renal disease (ESRD) patient and undoubtedly warrants extensive future study with a view to improved quality of life in the growing dialysis population.

## Introduction

Conventional hemodialysis (HD) is the most widely used renal replacement therapy (RRT) worldwide [1]. It is based on the diffusive transport of solutes through a semi-permeable membrane and is effective in removing small uremic toxins, correcting acid-base balance, and managing fluid and electrolyte balance [1]. However, it is not suitable for the effective removal of middle molecules, which are responsible for many of the complications of chronic kidney disease (CKD). This necessitates the introduction of alternative therapies such as online hemodiafiltration (OL-HDF), which provides clearance of these molecules through convection. Despite technological advancements and more than 40 years since its introduction as a RRT, the actual benefits of OL-HDF, even with high convective volume (Qo) usage, remain a subject of debate [1]. Three major randomized controlled trials have compared survival outcomes in patients receiving HD or post-dilution OL-HDF, reporting conflicting results [2-4]. Meta-analyses of the published studies have also been unable to provide a clear and definitive answer regarding the potential benefits of choosing one treatment over the other [1,5-9]. Overall mortality, anemia, phosphate control, and small molecule clearance appear to be insufficiently influenced by the treatment method. On the other hand, cardiovascular mortality, hemodynamic stability, and clearance of middle and protein-bound molecules seem to be better in patients treated with OL-HDF [1]. The currently available evidence is still not strong enough to prove that OL-HDF is associated with better clinical outcomes compared to conventional HD, and medicine is a science based on evidence.

The aim of the current study is to investigate the effect of OL-HDF and applied Qo on the clinical outcome in two groups of patients undergoing HD and OL-HDF by evaluating the indicators of dialysis adequacy (single-pool Kt/V (spKt/V), urea reduction ratio (URR%)), nutritional status (normalized protein catabolic rate (nPCR), serum albumin), control of the anemic syndrome (serum hemoglobin, administered average weekly dose of erythropoietin-stimulating agent (ESA)), as well as the achieved hemodynamic stability during the procedures and to evaluate its impact on the achieved quality of life and the prognosis of the patient. This is necessary due to the still-too-high mortality in the ever-growing population of HD patients, over 850 million people worldwide, which necessitates the urgent need to develop new strategies and technologies in HD treatment, as well as the routine implementation of convective therapies in clinical practice.

## Materials and methods

This is a retrospective study conducted at the Clinic of Nephrology and Dialysis, University Hospital "St. Marina", Varna, Bulgaria, for a two-year period from the beginning of January 2020 to the end of December 2021, during which the medical documentation and routine laboratory tests of 41 patients meeting the study criteria were examined.

Inclusion criteria for the study are that the patients have reached 18 years of age, have been on chronic dialysis treatment for a period of more than six months with depleted residual renal function, have signed an informed consent to participate in the study, and are with corrected iron deficiency. Exclusion criteria are patients under 18 years of age, those who have not signed an informed consent to participate in the study, those who do not have uncorrected iron deficiency, those who have active bleeding of any origin, and patients with a proven malignant process.

The studied patients were divided into two groups: Group 1 ((n=19) performing conventional HD with low-flux polysulfone dialyzers type Asahi) and Group 2 ((n=22) performing OL-HDF in post-dilution mode with high-flux dialyzers type F70 and applied high Qo (Qo>20 l/session)). Both groups of patients were administered dialysate flow rate (Qd)-Qd=500 ml/min and blood flow rate (Qb)-Qb=300±42 ml/min with an average weekly dialysis time of 12±0.13 hours. In the second year, in eight of the patients on OL-HDF (Group 2), Qo was changed to Qo<20 l/session, keeping the other conditions. For the purposes of the study, the above parameters were evaluated, also taking into account the frequency of episodes of intradialytic hypotension (IDH), as well as the time to recovery reported by the patients. The devices used for dialysis were Fresenius Medical Care, series 4008 and 5008 (Fresenius Medical Care AG & Co, Germany).

Laboratory tests for blood counts and biochemistry (urea, creatinine, and serum albumin) in all the studied patients are performed routinely, according to the requirements of the National Health Insurance Fund of the Republic of Bulgaria. Blood samples were collected under standardized conditions. Blood for hemoglobin analysis was drawn into K2EDTA tubes. Serum was obtained in a vacutainer with a gel separator and centrifuged for 15 minutes at 2500 G (6-8 ml). Serum was used for urea, creatinine, and albumin testing.

Hemoglobin concentration (g/l) was derived from the results of a complete blood count, using the colorimetric method with sodium lauryl sulfate on a 6-diff hematological analyzer Sysmex XN1000 (Siemens, Germany). Hemoglobin reference ranges (RRs) are 120-180 g/l. Ranges of 110-120 g/l are considered target values of hemoglobin concentration in patients with end-stage renal disease (ESRD).

Urea concentration (mmol/l) was measured by a coupled enzyme reaction with glutamate dehydrogenase (GLDH) using a UV kinetic method on the ADVIA Chemistry 1800 system (Siemens, Germany), with RRs of 3.2-8.2 mmol/l. Creatinine concentration (mmol/l) was measured using the Jaffe-kinetic method on the same system, with RRs of 44-115 mmol/l. Albumin concentration (g/l) was measured using a colorimetric method with the selective dye bromocresol green (BCG) on the ADVIA Chemistry 1800 system, with RRs of 32-48 g/l.

The stop pump technique was used before and/or after HD to avoid the effect of recirculation and minimize the effect of urea rebound.

Blood samples for serum hemoglobin and albumin were routinely taken before starting the dialysis procedure, thus avoiding the effect of applied ultrafiltration (UF). Blood samples for urea and creatinine were tested both before and at the end of the dialysis procedure to assess dialysis adequacy and nutritional status indicators: spKt/V-index, URR%, and nPCR. The latter were calculated on the basis of the formulas presented in Table 1.

**Table 1:** Mathematical methods spKt/V: single-pool Kt/V; URR%: urea reduction ratio; nPCR: normalized protein catabolic rate; TBW: total body water; t: dialysis time in hours (h); UF: the volume of ultrafiltration in liters (l); W: post-dialysis weight of the patient in kg; ln: natural logarithm; Co: pre-dialysis urea nitrogen; C: post-dialysis urea nitrogen; Cn: pre-dialysis urea nitrogen from the next hemodialysis; R: C/Co; ID: inter-dialysis time in hours (h); Age: age in years; Height: height in cm; Weight: optimal weight in kg

Indicator	Formula
spKt/V	spKt/V=​​-ln(R-0.008×t)+(4-3.5×R)×0.55UF/W
URR%	URR%=100×(1–C/Co)
nPCR	nPCR=0.22+0.36×(Cn-C)×24/ID
TBW men	TBW=2.447-0.09516×Age+0.1074×Height+0.3362×Weight
TBW women	TBW=-2.097+0.1069×Height+0.2466×Weight

Statistical analysis of the data was performed using IBM SPSS Statistics for Windows, V. 20.0 (Released 2011; IBM Corp., Armonk, NY, United States) with Windows 10 software (Microsoft Corporation, Redmond, WA, United States). The methods used include descriptive analysis to establish the average levels and variations in quantitative variables and absolute and relative values in qualitative variables, parametric methods for hypothesis testing (Student's t-test, analysis of variance (ANOVA), honestly significant difference (HSD) Tukey's post hoc test), receiver operating characteristic (ROC) curve analysis to establish the predictive significance of the studied quantities, and relative risk ratio analysis to establish the probability of an outcome in an exposed group. The results at p<0.05 are considered statistically significant.

## Results

The data from the variational analysis and Student's t-test for a two-year period are presented which show that there was a significant difference (p<0.05) in the studied indicators in the two groups, except for the registered levels of serum albumin in the second year of observation (t=1.20289, p=0.238) (Table 2). The results show significantly higher values of dialysis adequacy and nutritional status in patients with OL-HDF (Group 2), as well as higher values of serum hemoglobin in the group, achieved at significantly lower erythropoietin doses.

**Table 2:** Data from the variational analysis and Student's t-test for the two-year period of follow-up of the two studied groups spKt/V: single-pool Kt/V, dialysis adequacy index; URR%: urea reduction ratio, dialysis adequacy index; nPCR (g/kg/d): normalized protein catabolic rate; Alb (g/l): serum albumin; Hgb (g/l): hemoglobin; ESA (UI/week): erythropoietin stimulating agent

Indicator (X±SD)	Group 2 (n=22)	Group 1 (n=19)	T-test, p-value	Group 2 (n=21)	Group 1 (n=14)	T-test, p-value
Year	2020			2021		
Age (years)	53.9±11.96	56.63±11.07	t=-0.734, p=0.467	55.6±11.78	55.64±11.12	t=-0.738, p=0.465
spKt/V	1.82±0.12	1.3±0.11	t=14,52, p<0.00001	1.83±0.25	1.37±0.08	t=7.54, p<0.00001
URR%	79,46±1.45	69.01±5.8	t=7.647, p<0.00001	79.21±3.56	71.86±2.99	t=6.36, p<0.00001
nPCR (g/kg/d)	1.28±0.12	1.16±0.11	t=3.215, p=0.003	1.31±0.12	1.2±0.05	t=3.48, p=0.0024
Alb (g/l)	38.90±5.4	35.74±3.19	t=2.275, p=0.029	38.17±3.96	36.64±2.98	t=1.20289, p=0.238
Hgb (g/l)	111.64±4.43	102.32±5.19	t=6.20, p<0.00001	112.15±6.86	104.14±4.73	t=3.64, p<0.00001
ESA (UI/week)	5681±1742	9947±2328	t=6.53, p<0.00001	6142±2080	9714±1749	t=-4.01, p=0.000162

The results of the two studied groups in terms of applied UF, Qo, and episodes of IDH with the time required for recovery (recovery time (RT)) of the patients are presented in Table 3.

**Table 3:** The results of the two studied groups in terms of applied UF, Qo, and episodes of IDH with the time required for recovery of the patients UF (l): volume of ultrafiltration; Qo (l): convective volume; RT (h): recovery time; IDH (%): intradialytic hypotension

Indicator (X±SD)	Group 2 (n=22)	Group 1 (n=19)	T-test, p-value	Group 2 (n=21)	Group 1 (n=14)	T-test, p-value
Year	2020			2021		
UF (l)	3905±413	4042±414	t=-1.06, p=0.295	3890±274	3743±408	t=1.146, p=0.265
Qo (l)	24.73±0.98	-	-	22.57±4.31	-	-
RT (h)	2.86±2.61	5.79±4.52	t=-2.58, p=0.014	1.53±0.59	5.57±5.78	t=-2.51, p=0.026
IDH (%)	27%	42%	t=-0.986, p=0.165	4.8%	35.8%	t=-1.753, p=0.043

The obtained results show significantly shorter RT in the OL-HDF group (Group 2) compared to the patients with HD (Group 1), and although in the first year of observation the difference was not statistically significant (t=-0.986, p=0.165), at the end of the study, it demonstrated the pronounced effect of convective therapies on hemodynamics (t=-1.753, p=0.043) with secondary improved RT. This effect is a result of the correction of the applied Qo, which stands out as the main factor determining the clinical outcome (Table 4).

**Table 4:** Frequency of IDH after Qo correction Qo (l): convective volume; RT (h): recovery time; IDH (%): intradialytic hypotension

OL-HDF (X±SD)	2020 (n=22)	2021 (n=21)	T-test, p-value
Qo (l)	24.73±0.98	22.57±4.31	t=2.23053, p=0.015623
RT (h)	2.86±2.61	1.53±0.59	t=2.29736, p=0.013388
IDH (%)	27%	4.8%	t=1.74939, p=0.044257

For a more detailed analysis, the studied patients were conditionally divided into three equal intervals (tertiles) between the youngest (age 32) and the oldest (age 79) patients: T1 (32-47 years), T2 (48-63 years), and T3 (64-79 years). The data from the ANOVA by age are presented in Table 5. Patients in the upper tertile (T1) have a significantly higher frequency of IDH when applying a high Qo (Qo>20 liters) (t=-3.03489, p=0.004457). Reduction of the latter (t=4.04289, p<0.0001) in these patients leads to improved tolerability of the procedure and shortened RT, which is at the expense of the achieved clinical result (Table 6). The results of the ANOVA and HSD Tukey's post hoc test did not reveal a statistically significant difference (p>0.05) in the above indicators between the first and second years of the study in the other age groups (T1 and T2) of OL-HDF (Group 2). This has not been proven for any age group within the conventional HD (Group 1). It should not be neglected that after the correction of Qo, the obtained average values for spKt/V (t=1.37, p=0.09) and URR (t=1.43, p=0.08) in the third tertile (T3) are completely comparable with those in the same age group. Conducting HD (Group 1), a significant difference was found only in the parameters of nutritional status, nPCR (t=-2.59, p=0.01) and serum albumin (t=-6.64, p<0.001), which are significantly higher in this group, as the latter is also associated with better hemodynamic stability and RT in patients in the third age tertile (Table 7).

**Table 5:** Mean values of indicators in the two studied groups in the three tertiles (T1, T2, and T3) spKt/V: single-pool Kt/V, dialysis adequacy index; URR%: urea reduction ratio, dialysis adequacy index; nPCR (g/kg/d): normalized protein catabolic rate; Alb (g/l): serum albumin; Hgb (g/l): hemoglobin; ESA (UI/week): erythropoietin stimulating agent; Qo (l): convective volume; IDH (%): intradialytic hypotension; RT (h): recovery time; HD: hemodialysis

Indicator (X±SD)	Т1 (32-47 years)	Т2 (48-63 years)	Т3 (64-79 years)	Т1 (32-47 years)	Т2 (48-63 years)	Т3 (64-79 years)
Year	2020			2021		
Therapy	Online HDF					
spKt/V	1.84±0.10	1.9±0.12	1.73±0.04	2.0±0.10	1.96±0.08	1.57±0.24
URR%	79.55±0.82	80.15±2.02	79.01±0.72	81.16±1.13	81.46±1.86	75.73±3.13
nPCR (g/kg/d)	1.38±0.04	1.34±0.07	1.14±0.07	1.4±0.06	1.36±0.07	1.17±0.05
Alb (g/l)	42.53±0.60	41.35±4.18	32.9±3.37	42.48±2.02	39.65±2.43	33.98±7.73
Hgb (g/l)	112.5±4.03	112.8±4.83	109.75±4.65	111.4±6.84	116±6.27	108.5±6.54
ESA (UI/week)	4666±1032	4666±1032	7000±1511	4800±1095	4625±1767	8500±3338
Qo	24.5±1.22	24.6±1.03	24.8±0.99	25±0.70	25.37±0.74	18.25±4.52
IDH	0%	0%	75%	0%	0%	12.5%
RT (h)	1.16±0.40	1.5±0.54	5.5±2.72	1.12±0.21	1.25±0.46	2.13±0.35
Therapy	Conventional HD					
spKt/V	1.35±0.07	1.27±0.11	1.28±0.13	1.39±0.03	1.34±0.09	1.41±0.07
URR%	71.4±3.97	67.16±6.61	69.48±5.98	72.84±2.27	70.02±3.12	73.26±2.83
nPCR (g/kg/d)	1.21±0.02	1.10±0.10	1.21±0.11	1.2±0.01	1.16±0.05	1.25±0.05
Alb (g/l)	36.98±1.23	33.68±2.85	37.43±3.5	37.28±1.3	33.88±3.15	39.28±0,33
Hgb (g/l)	105.6±6.58	98.5±2.07	104.6±3.98	104.4±3.84	101.8±4.6	108.4±6.51
ESA (UI/week)	8400±2509	11125±1642	9666±2658	9200±1643	10600±1949	9000±1732
IDH	0%	87%	16.5%	20%	90%	4%
RT (h)	3.8±2.68	7.87±5.30	4.66±3.98	4.0±2.23	9.8±8.67	2.2±0.44

**Table 6:** Comparative analysis of the achieved result in the patients in the third tertile (T3) (64-79 years of age) from Group 2 for a two-year follow-up period (2020 and 2021) Qo (l): convective volume; IDH (%): intradialytic hypotension; RT (h): recovery time; spKt/V: single-pool Kt/V, dialysis adequacy index; URR%: urea reduction ratio, dialysis adequacy index; nPCR (g/kg/d): normalized protein catabolic rate; Alb (g/l): serum albumin; Hgb (g/l): hemoglobin; ESA (UI/week): erythropoietin stimulating agent

Indicator (X±SD)			T-test, p-value
Year	2020	2021	
Qo (l)	24.87±0.99	18.25±4.52	t=-4.04289, p<0.0001
IDH	75%	12.5%	t=-3.03489, p=0.0044
RT (h)	5.5±2.72	2.12±0.35	t=3.4733, p=0.002
spKt/V	1.73±0.04	1.57±0.24	t=1.7975, p=0.04
URR%	79.01±0.72	75.73±3.13	t=-2.877, p=0.006
nPCR	1.14±0.07	1.17±0.05	t=-0.979, p=0.17
Alb (g/l)	32.9±3.37	33.98±7.73	t=0.80, p=0.218
Hgb (g/l)	109.75±4.65	108.5±6.54	t=0.44, p=0.333
ESA (UI/week)	7000±1511	8500±3338	t=1.15, p=0.13

**Table 7:** Comparative analysis of the results achieved in patients of the two studied groups in the third tertile (T3) (64-79 years of age) for the two-year follow-up period (2020 and 2021) Qo (l): convective volume; IDH (%): intradialytic hypotension; RT (h): recovery time; spKt/V: single-pool Kt/V, dialysis adequacy index; URR%: urea reduction ratio, dialysis adequacy index; nPCR (g/kg/d): normalized protein catabolic rate; Alb (g/l): serum albumin; Hgb (g/l): hemoglobin; ESA (UI/week): erythropoietin stimulating agent

Indicator (X±SD)	Group 2	Group 1	T-test, p-value	Group 2	Group 1	T-test, p-value
Year	2020			2021		
Qo (l)	24.87±0.99	-	-	18.25±4.52	-	-
IDH	75%	16.5%	t=2.44, p=0.015	12.5%	4%	t=0.77, p=0.23
RT (h)	5.5±2.72	2.66±1.98	t=3.483, p=0.018	2.12±0.35	2.2±0.44	t=-0.33, p=0.37
Kt/V	1.73±0.04	1.28±0.13	t=8.61, p<0.0001	1.57±0.24	1.41±0.07	t=1.37, p=0.09
URR%	79.01±0.72	69.48±5.98	t=4.52, p=0.0003	75.73±3.13	73.26±2,83	t=1.43, p=0.08
nPCR	1.14±0.07	1.21±0.11	t=-1.35, p=0.10	1.17±0.05	1.25±0,05	t=-2.59, p=0.01
Alb (g/l)	32.9±3.37	37.43±3.5	t=-2.43, p=0.015	33.98±7.73	39.28±0,33	t=-6.64, p<0.001
Hgb (g/l)	109.75±4.65	104.6±3.98	t=2.14, p=0.026	108.5±6.54	108.4±6,51	t=0.02, p=0.48
ESA (UI/week)	7000±1511	9666±2658	t=-2.38, p=0.017	8500±3338	9000±1732	t=-0.3, p=0.38

The obtained results also show significant differences in the annual mortality rates for 2020 (Group 2: 4.5% and Group 1: 26% (χ²=18.478, V=0.521, p=0.001)) and for 2021 (Group 2: 14.3% and Group 1: 42.8% (χ²=13.147, V=0.513, p=0.001)). It was found that the relative risk of death in Group 1 is 3.6 times higher (RR: 3.59; 95% CI: 1.2531-10.2467, p<0.001) compared to Group 2. At the end of the period, the registered two-year survival in patients from Group 2 was 87.7%, while in Group 1, it was significantly lower at 57.2%.

**Figure 1:**
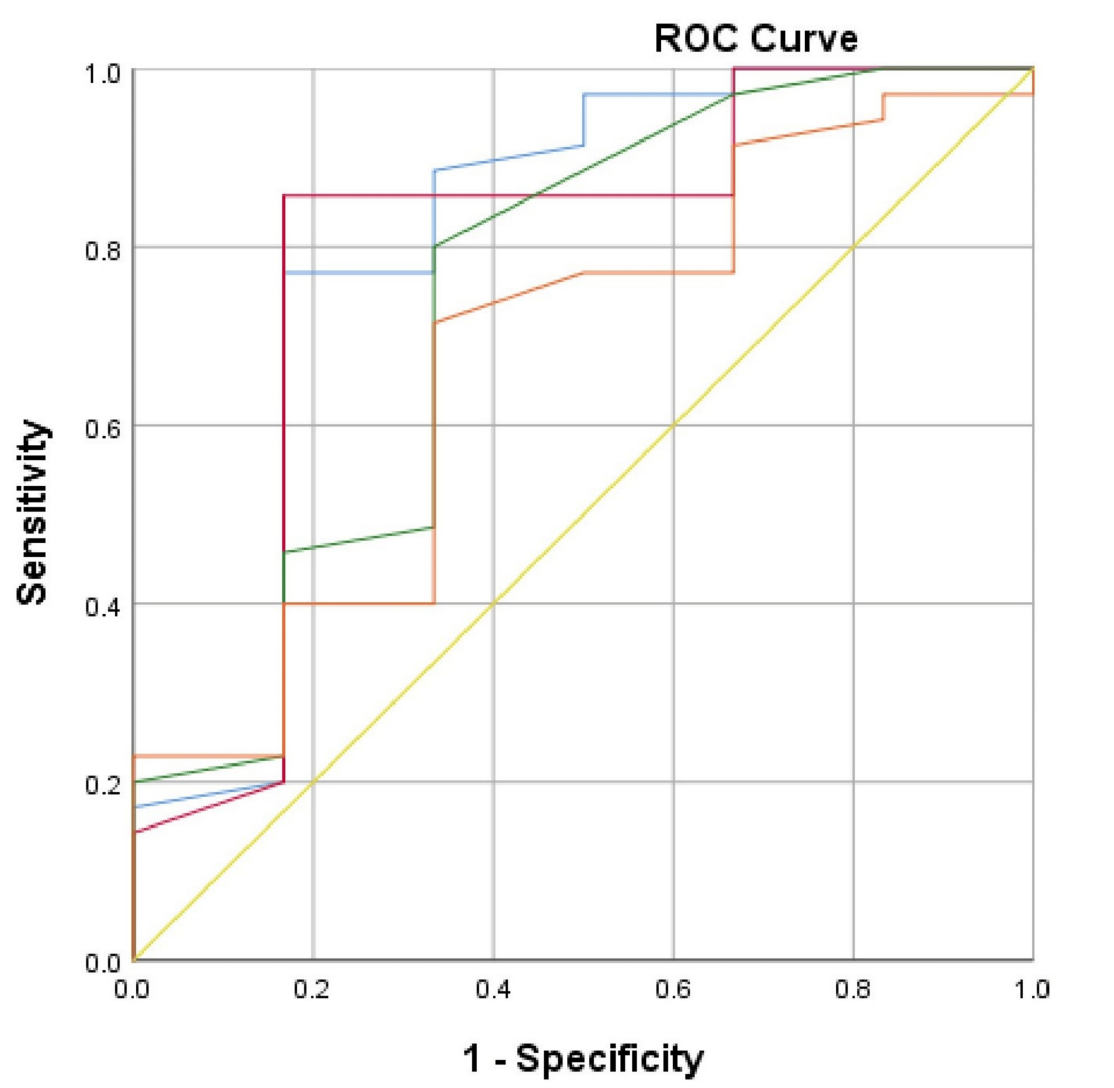
ROC curve for estimating the predictors of deteriorating clinical outcome of the patients from Group 1 ROC: receiver operating characteristic; blue line: single-pool Kt/V, dialysis adequacy index (spKt/V); red line: urea reduction ratio, dialysis adequacy index (URR%); green line: normalized protein catabolic rate (nPCR); orange line: serum albumin; yellow line: reference line

**Figure 2:**
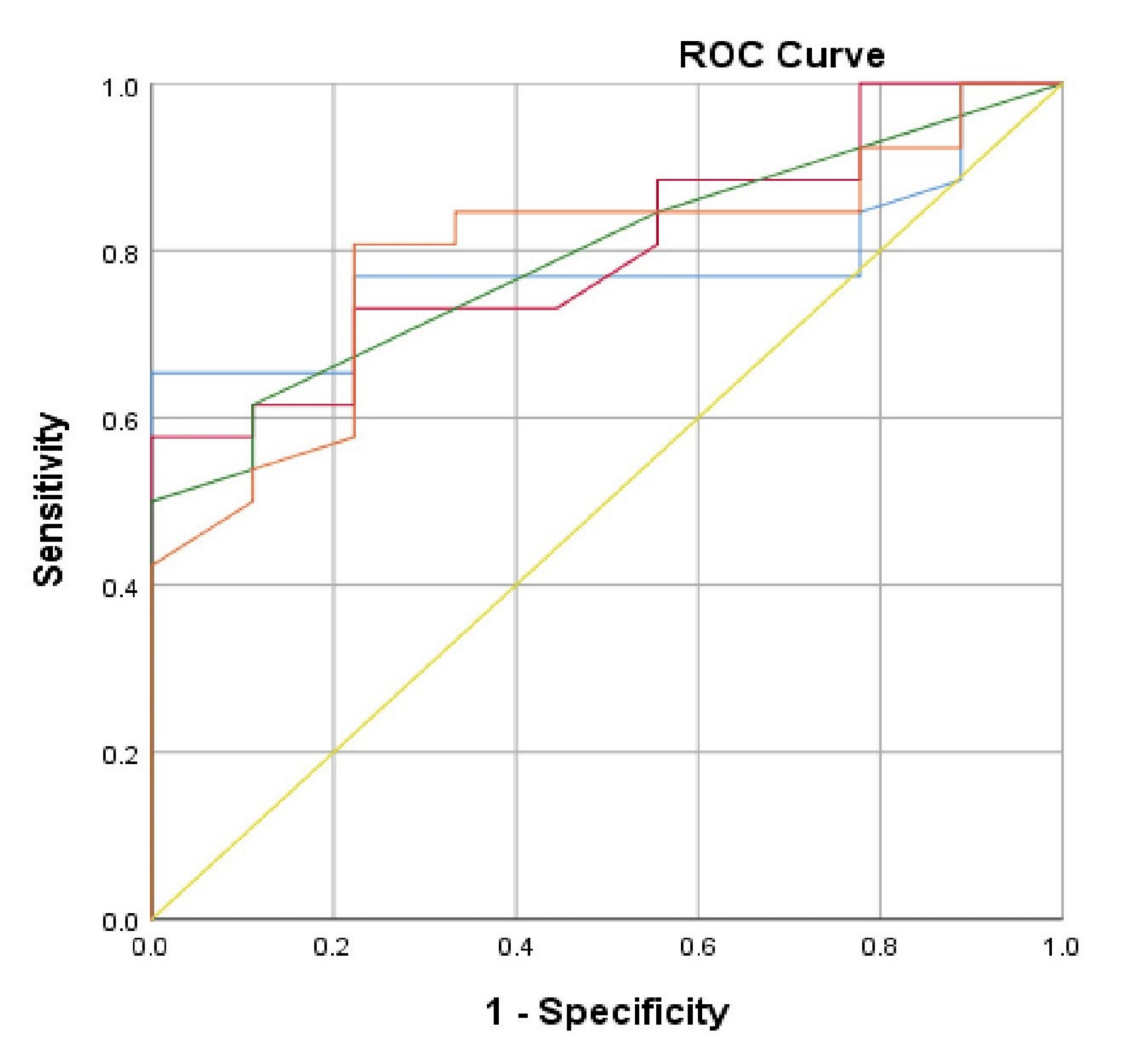
ROC curve for estimating the predictors of deteriorating clinical outcome of the patients from Group 2 ROC: receiver operating characteristic; blue line: single-pool Kt/V, dialysis adequacy index (spKt/V); red line: urea reduction ratio, dialysis adequacy index (URR%); green line: normalized protein catabolic rate (nPCR); orange line: serum albumin; yellow line: reference line

The ROC curves for determining the main predictors of deteriorating clinical outcome and death in the two studied groups for the two-year follow-up period are shown in Figure 1 and Figure 2.

ROC curve analysis demonstrates that in Group 1, the main predictors of deteriorating clinical outcome are spKt/V (AUC=0.805, 95% CI: 0.567-1.00, p=0.018) and URR (AUC=0.790, 95% CI: 0.552- 1.00, p=0.024) with critical values, respectively, spKt/V<1.25, with a sensitivity of 88.6% and a specificity of 33.3%, and URR <70.25%, with a sensitivity of 85.7% and a specificity of 16.7%. nPCR and serum albumin did not show statistical significance as predictors of death in our sample. The results of the ROC curve analysis in Group 2 show that the main predictors of deteriorating clinical outcome are both the dialysis adequacy indices, spKt/V (AUC=0.780, 95% CI: 0.629-0.931, p=0.013) and URR% (AUC=0.799, 95% CI: 0.652-0.946, p=0.008), and the indicators of nutritional status, nPCR (AUC=0.793, 95% CI: 0.644-0.941, p=0.010) and serum albumin (AUC=0.793, 95% CI: 0.637-0.949, p=0.010). The registered critical values for the studied indicators in the sample are spKt/V<1.4 (sensitivity 76.9%; specificity 22.2%), URR<74.4% (sensitivity 73.1%; specificity 22.2%), nPCR<1.2 (sensitivity 61.5%; specificity 11.1%), and serum albumin below 34.75g/l (sensitivity 84.6%; specificity 33.3%). Despite its high negative predictive value, the lowest sensitivity of the studied indicators is nPCR. Given the negative predictive value of nPCR and serum albumin and the data from the ANOVA with age, caution should be exercised in patients of the third age group (T3) performing OL-HDF (Group 2).

## Discussion

The results of our two-year follow-up demonstrate significantly higher values of the studied indicators of dialysis adequacy and nutritional status in patients undergoing OL-HDF (Group 2). The difference was not only observed for serum albumin levels at the end of the study, which was probably associated with loss during the procedure. A positive effect in Group 2 was also noted for the anemic syndrome with improved hemoglobin levels at reduced erythropoietin dose. It is noteworthy that the recorded mean values of spKt/V=1.82±0.12 and URR=79.46±1.45 are significantly higher in Group 2 and although these characteristics are representative of the purification of low molecular weight molecules, and not the medium molecules to which OL-HDF is directed, the results obtained by us demonstrate a significant effect in terms of diffusion clearance, which is consistent with the reports of Piccoli et al. from 2018 [10]. According to Cornelis et al., the clearance of urea and small molecules (<500 Da) is slightly higher in OL-HDF and should not be the only reason for choosing OL-HDF as a method for RRT [11]. The authors did not find a significant difference in the removal of serum phosphates [11].

Regarding the nutritional status in patients from Group 1, we recorded acceptable nPCR values according to the Kidney Disease Outcomes Quality Initiative (KDOQI) (2015) with a range of 1.16 at the beginning to 1.2 at the end of the study. However, we found significantly higher values in Group 2: nPCR=1.28 at the first year and nPCR=1.31 at the end of the observation. This, in our opinion, is due to the increased clearance of uremic toxins, not only low molecular weight but also medium molecules (500-60000 Da), as well as some of the serum protein, which significantly improves appetite and gastrointestinal manifestations of uremia. The results recorded by us for Group 2 are in contradiction with the data of Piccoli et al. from 2018, which report significantly lower values for nPCR in patients with OL-HDF, corresponding to deteriorating nutritional status [10]. We report the same discrepancy in the results with respect to serum albumin levels, which in our study were significantly higher than reported. However, it should be borne in mind that the study by Piccoli et al. is aimed at elderly patients [10]. When adjusting our results for age, we found that in the upper age tertile (T3), patients with OL-HDF tend to have poor nutritional status with nPCR<1.2 and moderately decreased serum albumin, which is confirmed by the above study [10]. This is most likely due to the loss of albumin during OL-HDF, which is a well-known effect of the procedure. The clinical effect of albumin loss is usually negligible and is offset by patients' improved appetite along with hepatic protein synthesis, but in elderly patients, it has a significant effect and should be considered as a potential risk. It should be borne in mind that the loss of albumin in OL-HDF is non-selective, i.e., not only the fraction associated with uremic toxins (so-called toxic albumin) is lost. According to some authors, the clearance of toxic albumin should be increased, but our opinion is that serum albumin levels below 35 g/l should be avoided [12]. It should not be overlooked that the loss of the latter is also a marker for the loss of other useful nutrients, including vitamins, and may further contribute to malnutrition and poor clinical outcome [12].

Opinions about the effect of OL-HDF on the control of anemia also remain conflicting. Our results demonstrate a strong correlation between the conducted convective therapy, both with the achieved hemoglobin levels, which are significantly higher than those in patients with HD (Group 1), and with the applied ESA dose, which shows a lasting tendency to decrease compared to conventional HD. This is probably due to the improved purification of medium molecular weight (500-60000 Da) erythropoiesis inhibitors, as well as the use of ultrapure dialysate produced by online technology. Unlike the parameters of the nutritional status, no reliable age dependence was found here. Several small uncontrolled studies as well as a small randomized trial confirm our results. They report an improvement in anemia control after switching from standard low-flux HD (LF-HD) to the use of high-permeability and biocompatible membranes in OL-HDF [1,13,14]. Ok et al. in a Turkish hemodiafiltration study from 2013 also reported that the applied dose of ESA was significantly lower in patients with OL-HDF, which is in line with our results, but nevertheless did not show a significant difference in relation to the achieved hemoglobin levels [1,4]. At the same time, data from Maduell et al. from 2013 from the Spanish study Estudio de Supervivencia de Hemodiafiltración Online (ESHOL) as well as a meta-analysis from 2013 of 65 studies comparing convective therapies with conventional HD did not show a significant improvement in serum hemoglobin levels and erythropoietin dose [1,8]. Similar data were reported by Schneider et al. in the Modulation of INflammation and OXidative stress by high-flux hemodialysIS (MINOXIS) study from 2012, as well as Panichi et al. in a randomized cross-over multicentre study (the REDERT study) from 2014, the latter reporting only a significantly higher administered ESA dose in patients with HD, which is consistent with our data [1,15,16]. Based on the results achieved, our opinion is that high-volume OL-HDF contributes to better control of renal anemia at lower erythropoietin doses. This is related on the one hand to the improved clinical outcome and quality of life of the patient and on the other hand to the reduced financial cost of erythropoietin therapy. On the other hand, the secondary effect of controlling renal anemia should not be neglected, namely, reduced cardiovascular risk and reduced incidence of associated hospitalization and death. Therefore, despite the higher cost of the procedure, the improved clinical outcome ultimately leads to a lower total cost of treatment.

There is a debate in the literature as to whether OL-HDF can improve hemodynamic stability. Some studies by Maduell et al. and Locatelli et al. demonstrate a beneficial effect of OL-HDF compared to HD in this regard [1,3,17,18]. Four meta-analyses from 2013 and 2014 also report the positive effect of convective techniques to improve hemodynamic stability, conclusions that are consistent with our results [1,5-9]. Our study demonstrated a significantly lower incidence of episodes of IDH with improved RT in patients undergoing OL-HDF, which is particularly pronounced at the end of the two-year follow-up period (p<0.05). Although in the first year the result was contradictory and no advantage of convective therapies was found in terms of hemodynamic stability (p>0.05), which was reported by some studies, we found that after adjusting the Qo according to individual characteristics of the individual, there is a significant reduction in episodes of IDH from 27% in 2020 to 4.8% in 2021 (t=1.74939, p=0.044257) and secondary improved RT [11,19,20]. Our study also found age dependence of tolerance to the procedure; patients in the upper age tertile (64-79 years of age) have difficulty tolerating high Qo, Qo>20 l, and respond with pronounced hemodynamic instability and prolonged RT, which is surprisingly not observed in the relevant age group. After correction and reduction of Qo on average to 16-18 liters per procedure, there is a significant reduction in the manifestations of IDH and shortening the recovery time from an average of 5.5±2.72 hours in 2020 to 2.12±0.35 hours in 2021 (p<0.05). This reflects on the clinical outcome, which becomes comparable to that of adult patients undergoing HD. In our opinion, this requires strict refinement and an individual approach in the choice of therapy for this fragile population of patients in whom the pursuit of high efficiency of therapy and improved clearance of uremic toxins can significantly impair quality of life through hemodynamic instability and prolonged recovery of the patients. Our results are inconsistent with some studies by Cornelis et al., Morena et al., and Smith et al., which do not demonstrate the benefits of OL-HDF on hemodynamic stability [11,19,20].

The data reported so far on the effect of OL-HDF on mortality are contradictory. Only one of five recent randomized studies (including three specifically designed to test mortality as a primary endpoint) showed better patient survival of OL-HDF compared to HD [2-4,17,19]. According to Grooteman et al. in the CONTRAST study (Convective Transport Study), the overall mortality rate was not affected by the type of therapy used, although subgroup analysis suggested the benefit of high-volume OL-HDF (Qo>20 l) (HR: 0.66; p=0.03) [1,2]. However, a post hoc analysis of the Turkish OL-HDF study showed that OL-HDF with Qo>17.4 l was associated with a 46% reduction in overall mortality (p=0.02) and a 71% reduction in cardiovascular mortality (p=0.003) compared to HD [1,4]. As already mentioned, ESHOL is the only randomized study that shows a significant advantage of OL-HDF in terms of overall mortality, mortality from cerebrovascular and cardiovascular events, and mortality related to infections, and this is the study with the highest achieved Qo: 22.9-23.9 l/session [3,18]. Our results, like those presented so far, demonstrate an association of OL-HDF with improved patient survival. There was a significant difference in the recorded overall mortality rates between the two groups, with the analysis showing a 3.6-fold higher risk of death (RR: 3.59; OR: 4.88; 95% CI: 1.2531-10.2467, p<0.001) in the conventional HD group. At the end of the period, the two-year survival rate in patients with OL-HDF was 87.7%, while in those with HD, it was significantly lower: 57.2%. Data from the ROC curve analysis of total mortality show that for the group of conventional HD, the main predictors of poor outcome and death were spKt/V<1.25 and URR<70.25%. In the OL-HDF group, the main predictors of deteriorating clinical outcome were both the dialysis adequacy indices and the indicators of nutritional status with critical values spKt/V<1.4, URR<74.4%, nPCR<1.2, and serum albumin <34.75 g/l. This clearly shows that in the conditions of high dialysis doses and convective therapies, the assessment of dialysis adequacy and risk stratification should always be complex with an emphasis on the parameters of nutritional status. Although serum albumin did not show statistical significance as a negative predictor of ROC curve analysis for Group 1, our opinion is that it should be interpreted as a proven independent risk factor for death in any periodic dialysis patient. Several meta-analyses by Susantitaphong et al., Nistor et al., and Wang et al. for the possible benefits of OL-HDF on patient survival do not prove an advantage of the studied convective therapies, both in terms of overall and in terms of cardiovascular mortality, and are in contrast to our results [6,8,9].

Limitations

It should not be overlooked that our study has some limitations due to its retrospective nature and small sample size (due to the small number of devices providing the study), which did not allow us to study the clearance of medium molecules such as beta-2 microglobulin, which is not routinely examined in clinical practice, and it is a future prospect for research. We were also limited in our follow-up period due to the significantly higher cost of consumables for OL-HDF, a procedure that is not reimbursed separately by the National Health Insurance Fund and was carried out on a goodwill basis within the general budget. Аccordingly, we selected a lookback period ensuring the representativeness of our sample.

## Conclusions

Despite the small number of studies and the inconsistency of the data currently reported, OL-HDF shows an advantage both in terms of clinical outcomes and in terms of patient prognosis. We are aware that despite the reported encouraging results as increased dialysis dose, improved nutritional status, control of anemic syndrome with reduced erythropoietin dose, and better hemodynamic stability, further studies are needed to clarify some of the unresolved issues outlined above, which are reported and in world literature. Of course, despite the seeming many benefits of OL-HDF, we realize that this is not an ideal therapy for every patient, which shows that we must put the individualized approach to medicine first. In our opinion, the choice of therapy in elderly patients should be strictly specified, because dialysis adequacy is not only embodied in the digital values of generally accepted indicators but is a much broader concept, and should ensure a balance between clinical goals and quality of the patient's life. At the same time, this does not mean that OL-HDF is only suitable for young patients. The choice of therapy should weigh very well the benefits and risks for the individual in the name of better clinical outcomes and not be based solely on the choice of "new" and "modern".
